# Supplementary material for: Phytochemical Characterization of Olea europaea L. Cultivars of Cilento National Park (South Italy) through NMR-Based Metabolomics
Source: Molecules. 2021 Jun 24;26(13):3845. doi: 10.3390/molecules26133845 (PMC8270249; doi:10.3390/molecules26133845)
Supplement: Supplementary file 1 [file molecules-26-03845-s001.zip › molecules-1265001-supplementary.pdf]

Phytochemical Characterization of *Olea europaea* L. Cultivars of Cilento National Park (South Italy) through NMR-Based Metabolomics

Assunta Esposito [1], Pietro Filippo De Luca[1], Vittoria Graziani[1], Brigida D'Abrosca[1], Antonio Fiorentino[1,*] and Monica Scognamiglio [1]

SUPPLEMENTARY MATERIAL

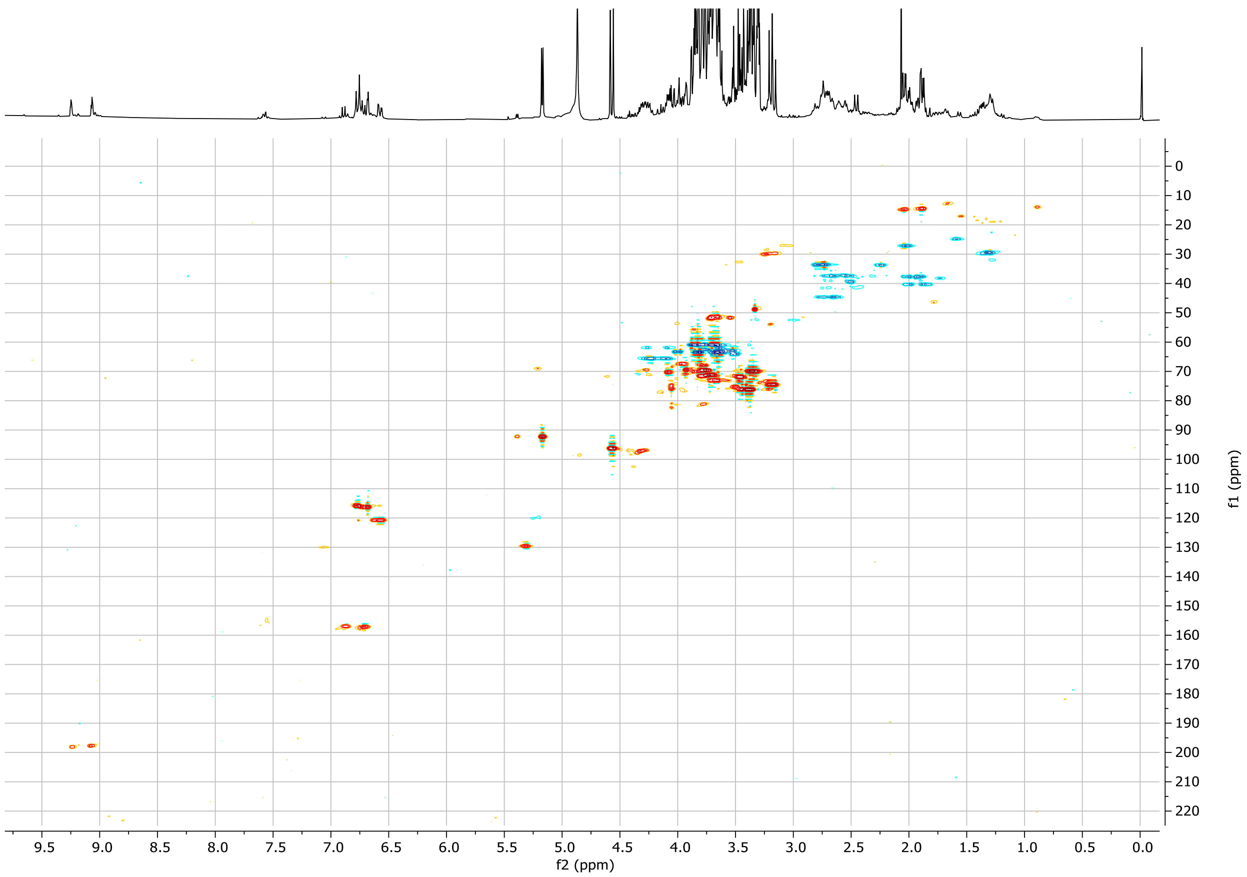

Figure S1. HSQC of Cammarotana cultivar

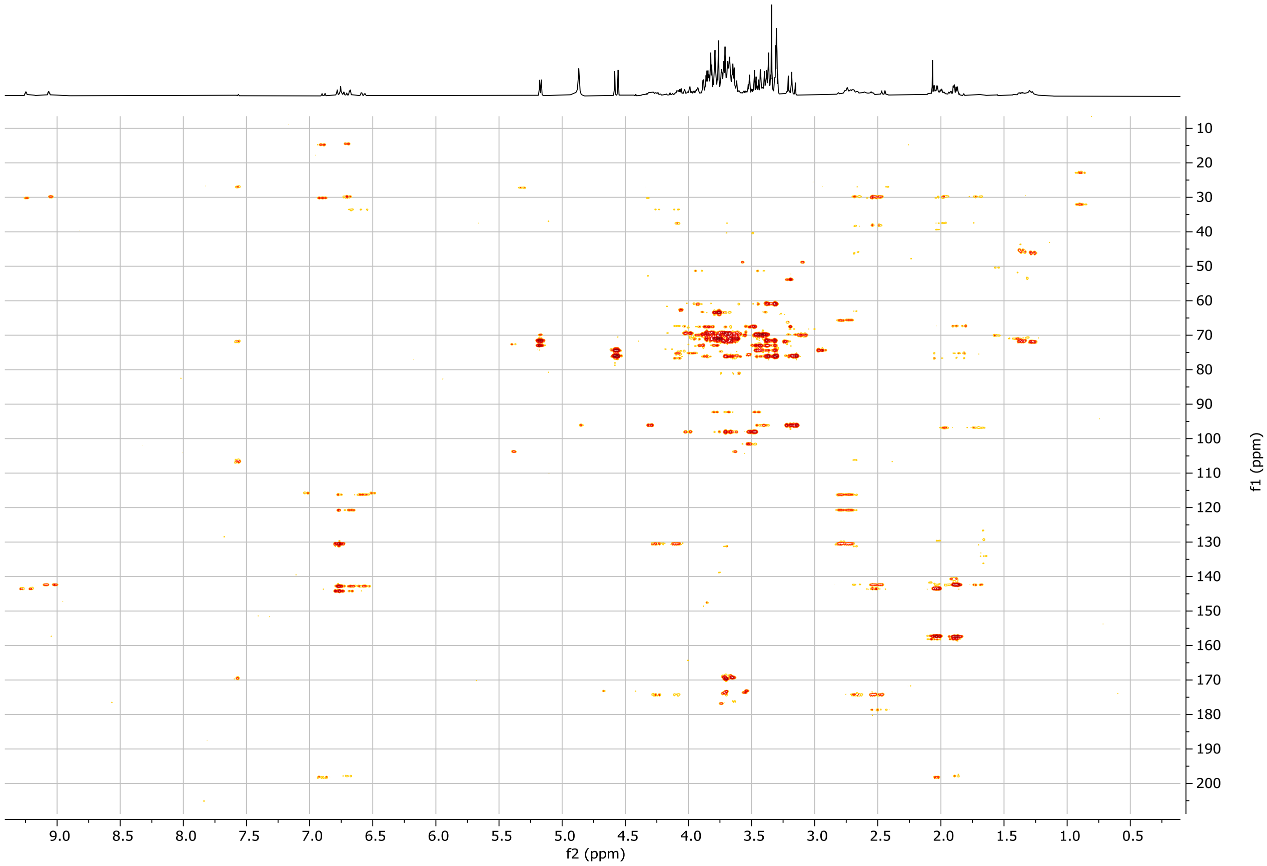

Figure S2. HMBC of Cammarotana cultivar

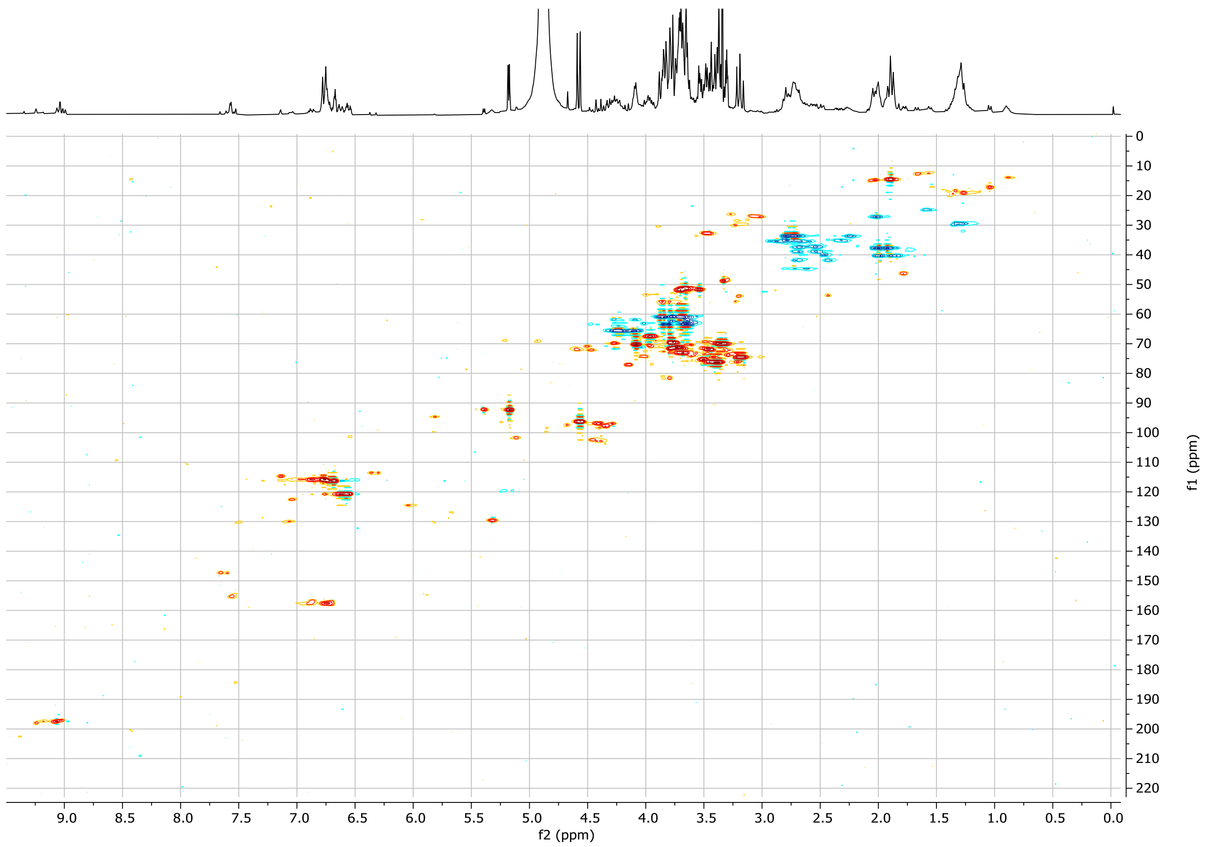

Figure S3. HSQC of Pisciottana cultivar

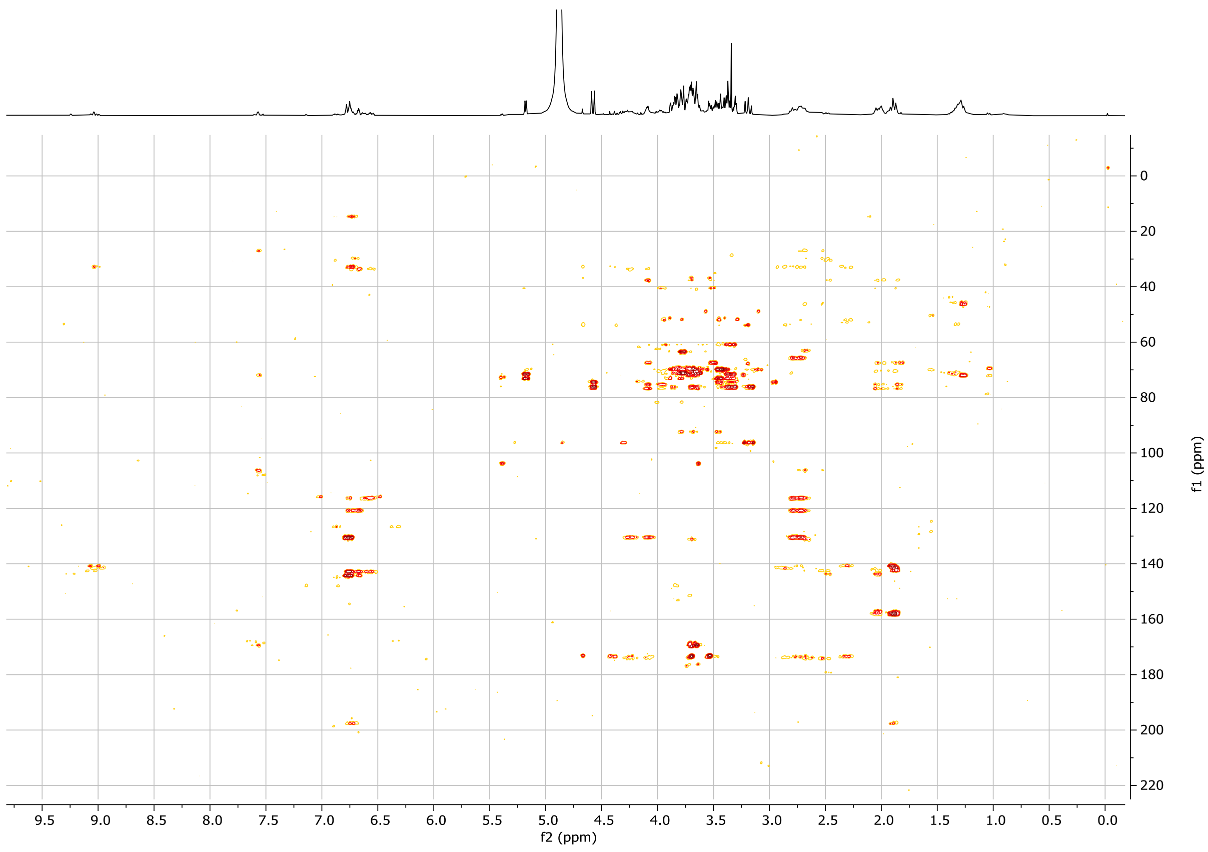

Figure S4. HMBC of Pisciottana cultivar

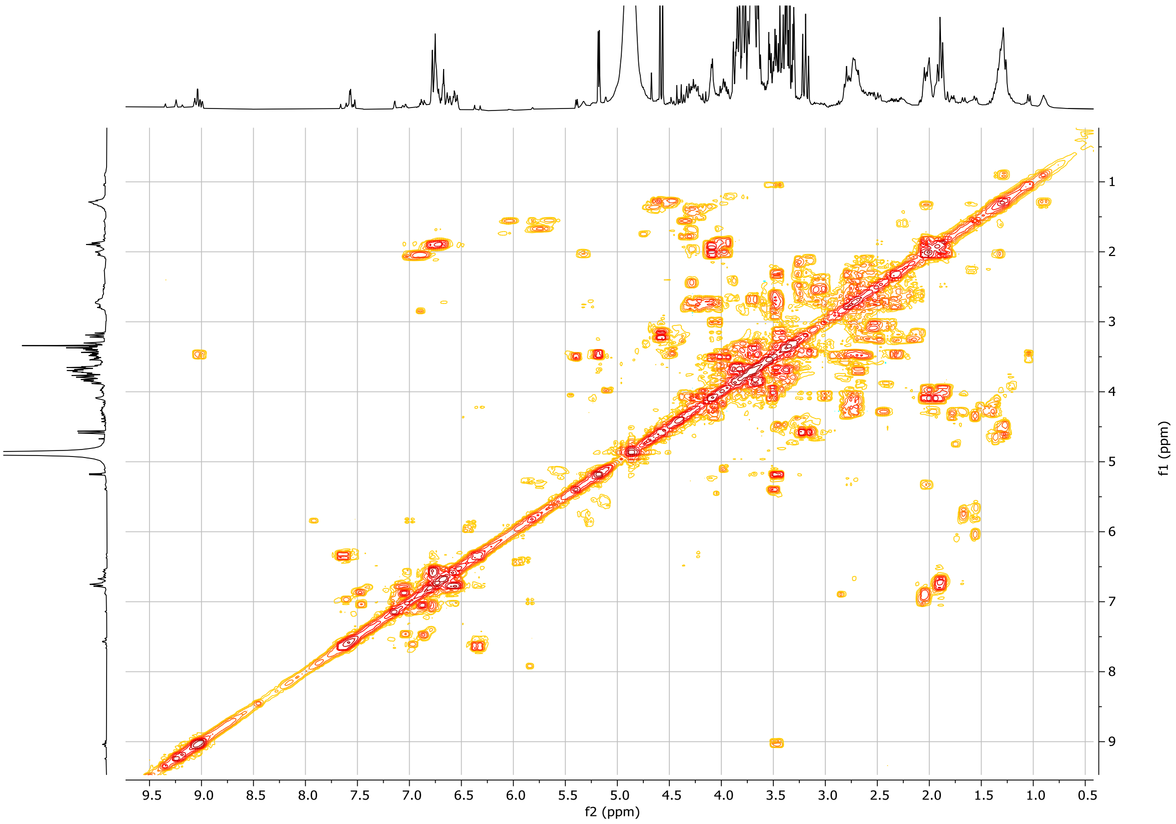

Figure S5. COSY of Pisciottana cultivar

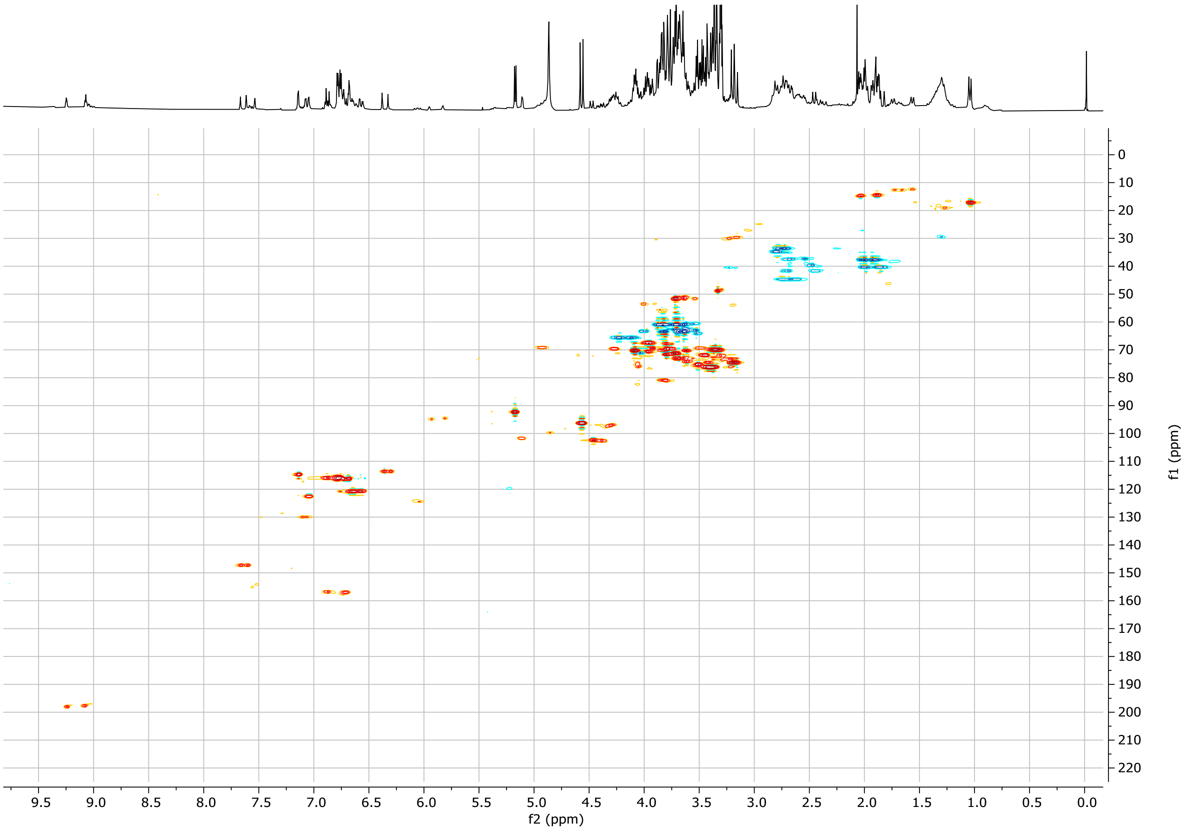

Figure S6. HSQC of Racioppa cultivar

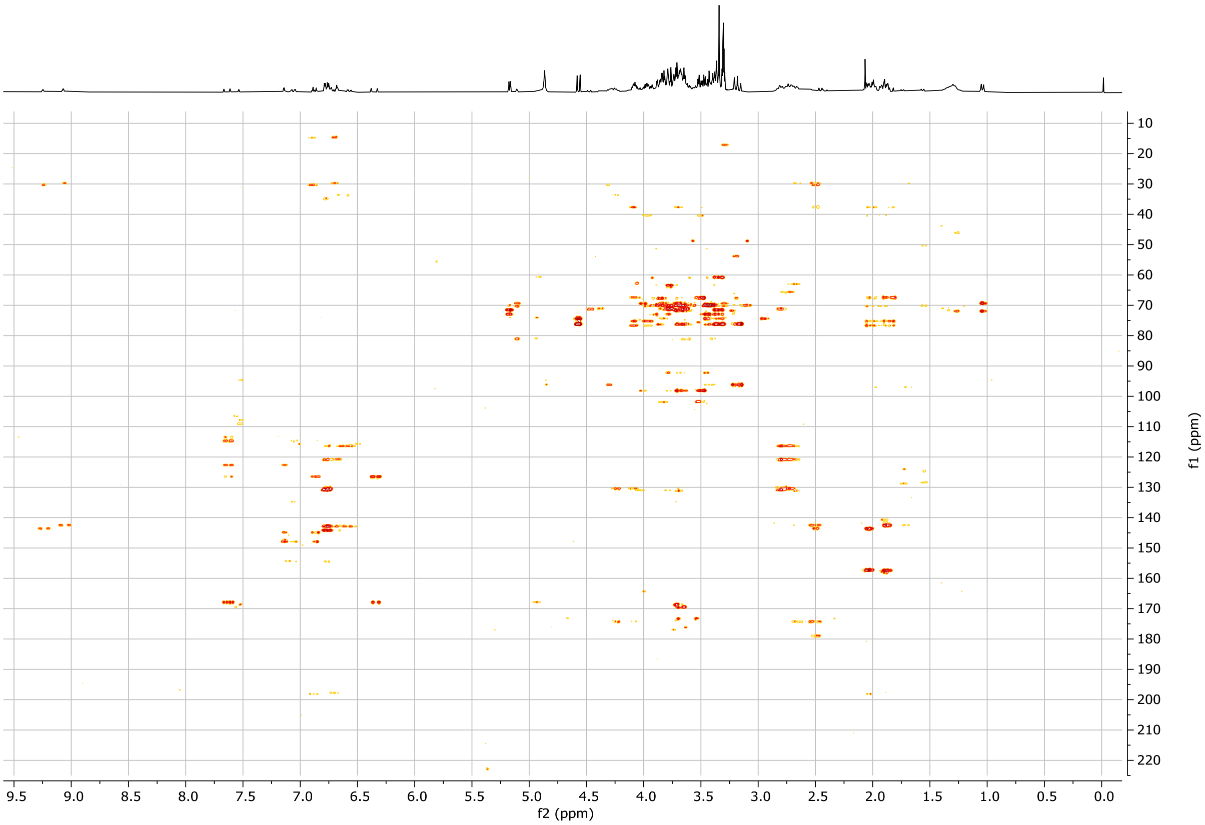

Figure S7. HMBC of Racioppa cultivar

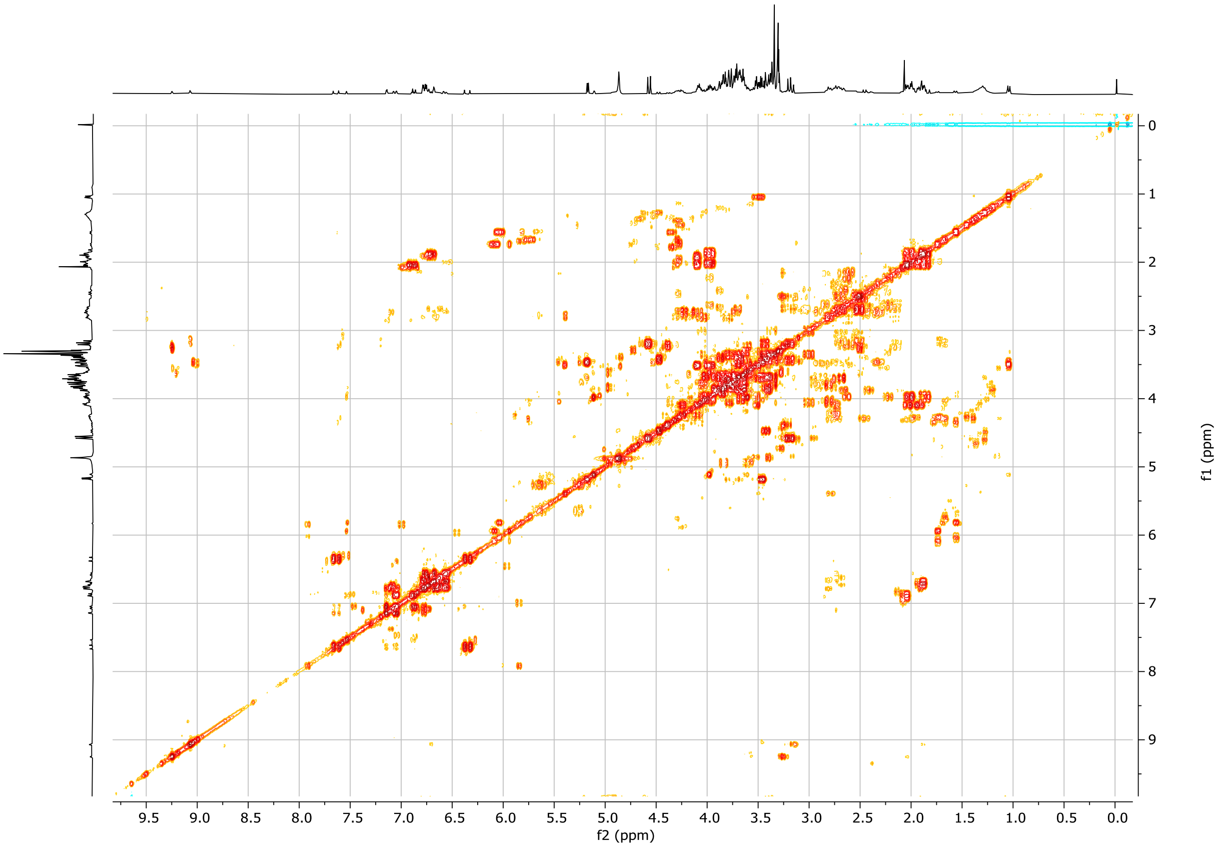

Figure S8. COSY of Racioppa cultivar

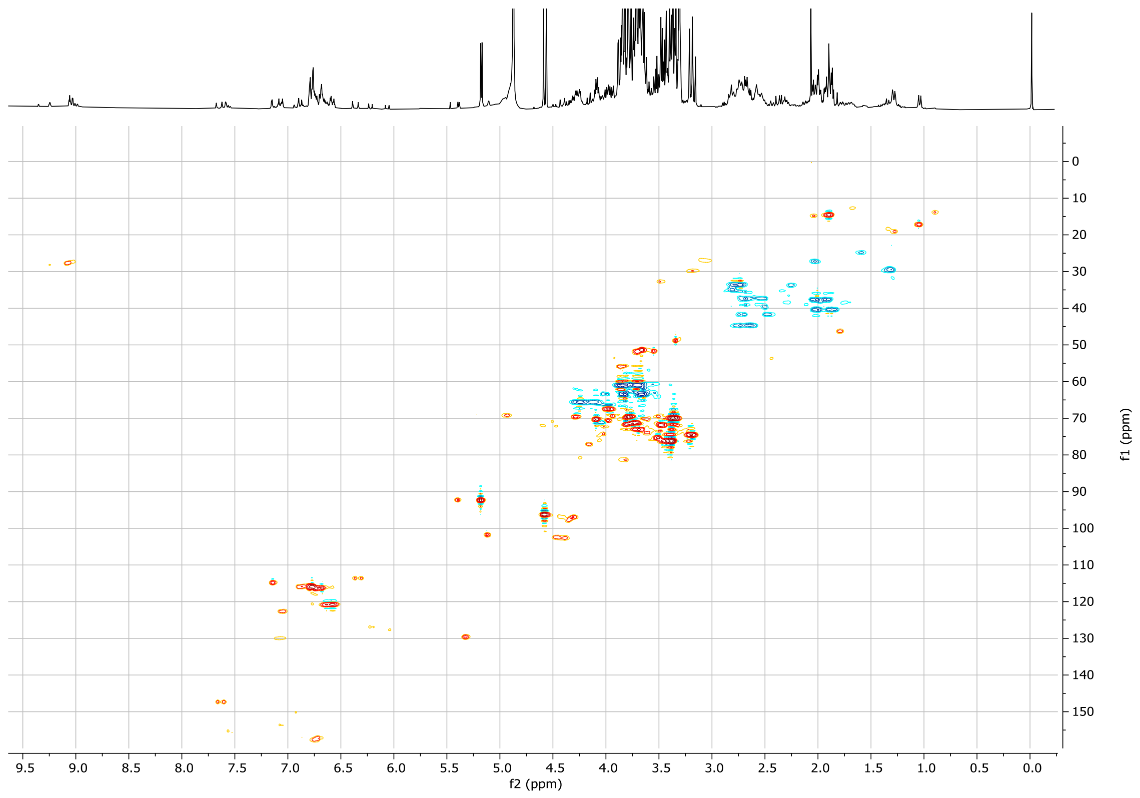

Figure S9. HSQC of Grossale cultivar

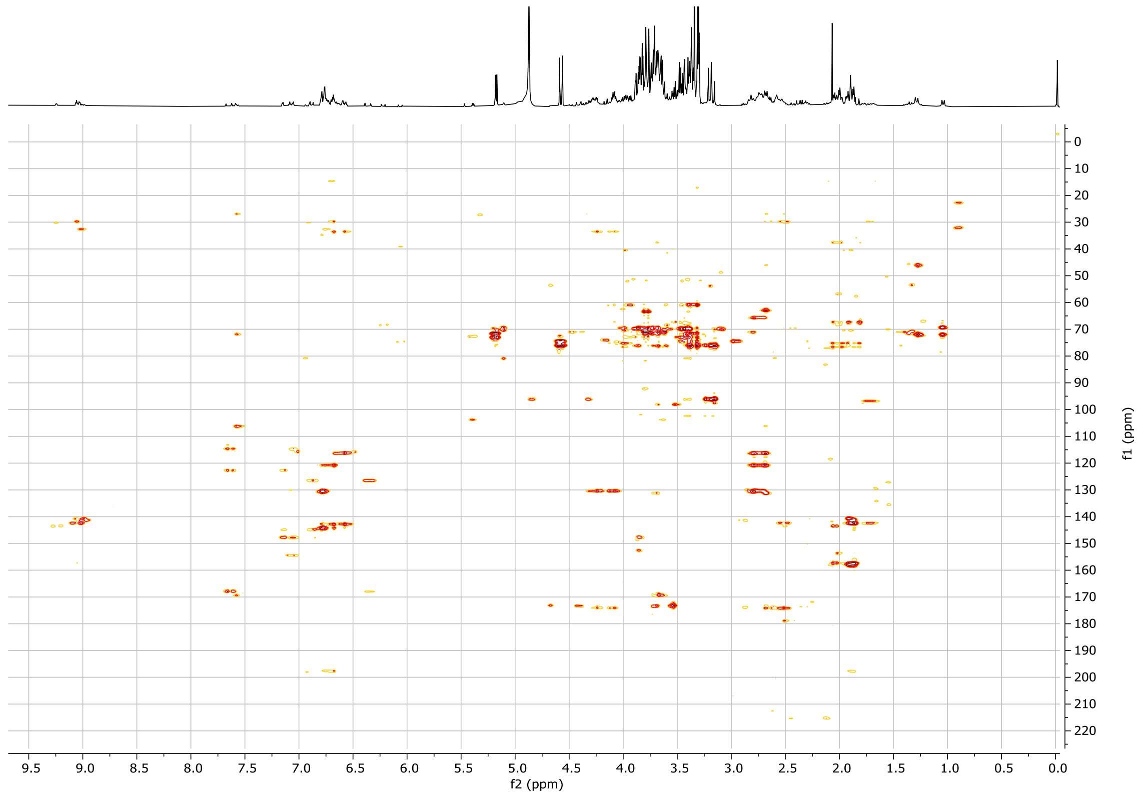

Figure S10. HMBC of Grossale cultivar
